# Factors associated with help-seeking behaviour among individuals with major depression: A systematic review

**DOI:** 10.1371/journal.pone.0176730

**Published:** 2017-05-11

**Authors:** Julia Luise Magaard, Tharanya Seeralan, Holger Schulz, Anna Levke Brütt

**Affiliations:** 1 Department of Medical Psychology, Center for Psychosocial Medicine, University Medical Centre Hamburg-Eppendorf, Hamburg, Germany; 2 Department of Health Services Research, Carl von Ossietzky University, Oldenburg, Germany; Iranian Institute for Health Sciences Research, ISLAMIC REPUBLIC OF IRAN

## Abstract

Psychological models can help to understand why many people suffering from major depression do not seek help. Using the ‘Behavioral Model of Health Services Use’, this study systematically reviewed the literature on the characteristics associated with help-seeking behaviour in adults with major depression. Articles were identified by systematically searching the MEDLINE, EMBASE and PsycInfo databases and relevant reference lists. Observational studies investigating the associations between individual or contextual characteristics and professional help-seeking behaviour for emotional problems in adults formally diagnosed with major depression were included. The quality of the included studies was assessed, and factors associated with help-seeking behaviour were qualitatively synthesized. In total, 40 studies based on 26 datasets were included. Several studies investigated predisposing (age (N = 17), gender (N = 16), ethnicity (N = 9), education (N = 11), marital status (N = 12)), enabling (income (N = 12)), need (severity (N = 14), duration (N = 9), number of depressive episodes (N = 6), psychiatric comorbidity (N = 10)) and contextual factors (area (N = 8)). Socio-demographic and need factors appeared to influence help-seeking behaviour. Although existing studies provide insight into the characteristics associated with help seeking for major depression, cohort studies and research on beliefs about, barriers to and perceived need for treatment are lacking. Based on this review, interventions to increase help-seeking behaviour can be designed.

## Introduction

Major depression is a common mental disorder and one of the leading causes of health impairment worldwide [1], resulting in serious impairment of functioning and decreased quality of life [2, 3]. To treat major depression depending on severity, American and European guidelines (e.g. [4, 5]) recommend treatment options as psychotherapy, pharmacotherapy, or a combination of both. Despite the availability of effective treatment options, researchers continue to find that a significant number of individuals suffering from major depression do not seek professional help. Using studies on service utilization rates for major depression in community-based surveys, Kohn, Saxena [6] reported that the percentage difference between number of people needing treatment for major depression and number of people seeking professional help ranged between 15.9% (12 month, Florence) [7, 8] and 83.9% (current, UK) [9]. They estimated that the median untreated rate for depression is 56.3% worldwide [6].

**Fig 1:**
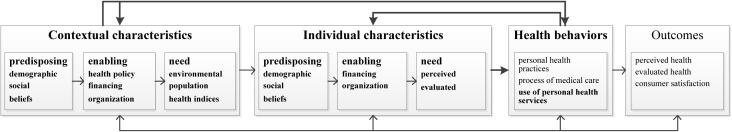
Behavioral model of health services use [15].

Various psychological models have been used to explain variations in help-seeking behaviour among populations, such as the Self-Regulation Model [10], the Health Belief Model [11] and the Theory of Planned Behavior [12]. From the sociological perspective models like the Pescosolido’s Network Episode Model [13], Kadushin’s theory about why people go to psychiatrists [14] and the Behavioral Model of Health Services Use [15] were specifically constructed to explain help-seeking behaviour. The ‘Behavioral Model of Health Services Use’ suggests that people’s predisposition to use services, factors which enable or impede the use of services and people’s need of care predict and explain health behaviours like use of health services [15]. According to the model, all health behaviours influence health related outcomes. The model includes feedback loops to demonstrate that outcomes can affect health behaviours, predisposing, enabling and need factors and health behaviours can influence predisposing, enabling and need factors. In the current version of his ‘Behavioral Model of Health Services Use’, Andersen [15] distinguishes between contextual and individual characteristics influencing service utilization and health-related outcomes (Fig 1). The model asserts that contextual and individual characteristics consist of predisposing, enabling and need factors [15]. Individual characteristics are measured at the individual level, whereas contextual characteristics are measured at an aggregate level (e.g., families, communities, national health care system). Contextual characteristics include health organizations and provider-related factors as well as community characteristics [15]. At the individual level, a person’s beliefs (e.g., attitudes towards health services), demographic characteristics (e.g., age) and social factors (e.g., education) define his or her predisposition to use health services. Additionally, the availability of financial resources to pay for services as well as organizational factors (e.g., regular source of care, means of transportation to care) enable or impede the use of health services at the individual level. In the “Behavioral Model of Health Service Use” it is not clearly defined if social relationships and social support are considered as predisposing or enabling factors. We agree with Andersen’s argumentation that social support can facilitate or impede help-seeking behaviour and therefore serves as an enabling resource [15] whereas the social structure including family situation predisposes help-seeking. Furthermore, perceived and evaluated need influences help-seeking behaviour. Professional judgement about people’s health and need for treatment is represented by evaluated need whereas perceived need includes people’s perspective on their own health [15]. The model has frequently used in studies and systematic reviews (e.g. [16, 17, 18]). According to validity, associations between different individual characteristics and services use were found empirically. However, causal conclusions cannot be drawn from analyses on the basis of mainly cross-sectional data (e.g. [16]). Individual characteristics of the current model can be expanded to include predictors of help-seeking behaviour like treatment and illness beliefs [10], perceived susceptibility and severity of symptoms as well as perceived expectations regarding treatment and self-efficacy [11, 12] and motivational factors [12].

The current review focusses on contextual and individual characteristics as well as use of personal health services and relations between characteristics and use of personal health services (printed in bold).

In recent years, several quantitative studies have used Andersen’s model to investigate the factors influencing professional help-seeking behaviour among individuals suffering from depression (e.g. [17, 18]). Additional quantitative studies on this subject have been conducted without referral to Andersen’s model (e.g. [19]). However, a systematic review of these findings has not been performed. The only existing review [20] was published 14 years ago and focused on studies using heterogeneous definitions of depression or depressive symptoms and help-seeking behaviour, finding that the help-seeking behaviour of individuals with depression or depressive symptoms was influenced by age, ethnicity, social support and clinical and psychiatric factors. Further studies focussed on specific populations [21] or specific factors associated to help-seeking [22, 23]. Recently, a qualitative synthesis of interview studies about help-seeking behaviour among people with depression was published [24].

The purpose of this review was to apply a theoretical framework to investigate the individual and contextual characteristics associated with professional help-seeking behaviour for emotional problems in adults with major depression. Therefore, the current review addresses two questions: (1) Which characteristics associated with help-seeking behaviour in adults suffering from major depression are investigated in the literature? (2) How are these characteristics related to help-seeking behaviour in adults suffering from major depression?

In addition to including new literature, this review expands upon previous reviews in two ways: first, it embeds the findings within the ‘Behavioral Model of Health Services Use’ framework and integrates aspects of different models. By systematically reviewing observational studies using standardized diagnostic instruments to assess major depression, this review aims to synthesize the results of studies assessing help-seeking behaviour in a homogeneous population.

## Methods

To the extent that they were applicable to observational studies and to the qualitative synthesis of results, the methods and results are reported in accordance with the Preferred Reporting Items for Systematic Reviews and Meta-Analyses (PRISMA) statement [25] (S1 Appendix) and the Meta-analysis of Observational Studies in Epidemiology (MOOSE) statement [26]. No review protocol exists.

### Search strategy

Two researchers (JLM, ALB) searched the MEDLINE, EMBASE and PsycInfo electronic databases in February 2017 (09.02.2017) using key words and a standardized vocabulary (e.g., MeSH) presented in S2 Appendix. These terms aimed to represent the concepts of ‘Depression’ and ‘Help-Seeking’. The search was restricted to ‘human’ and ‘English or German’. Additionally, the EMBASE search was restricted to ‘article’, and the search in PsycInfo to ‘all journals’.

### Study selection

After excluding double hits, the title and abstracts of all articles (published in English or German) identified through the electronic search were screened to exclude clearly irrelevant articles. Two researchers (TS, JLM) independently screened the title and abstracts of 150 records. If at least moderate agreement was achieved (Kappa ≥ .41) [27], the remaining records were screened by JLM. Additionally, the reference lists of the relevant studies and reviews identified in the electronic search were manually examined.

In the second step, the full texts of all potentially relevant studies were independently reviewed by two researchers (TS, JLM). The decision to include studies was based on a priori defined inclusion criteria (IC) (S3 Appendix).

### Study design

To identify the factors associated with help-seeking behaviour, we relied on observational quantitative studies because randomization of these influencing factors is not possible. Therefore, cohort, case-control and cross-sectional studies were included (IC 1), but intervention studies were excluded unless they retrospectively assessed help-seeking behaviour at baseline.

### Population

To investigate the factors of interest in a population with a comparable depression status, studies reporting on the help-seeking behaviour of individuals with a major depressive episode or major depression disorder were included (IC 2). To ensure the validity of the diagnoses, a sample or subsample with formally diagnosed major depression disorder or a major depression episode according to the Diagnostic and Statistical Manual of Mental Disorders (DSM), International Statistical Classification of Diseases (ICD) or Research Diagnostic Criteria (RDC) was required (IC 3). We included studies investigating adult populations (IC 4) with depressive subsamples of population-based datasets to ensure that the samples included individuals not seeking care (IC 5).

### Outcome

Based on the guidelines and in accordance with other reviews on help-seeking [16, 23, 28], we defined professional help-seeking as contacting a health practitioner or service for mental health reasons at least once or receiving therapy including primary care and specialized care in outpatient and inpatient settings in a defined time period (IC 6). To ensure the homogeneity of our outcome, we decided to exclude studies assessing lifetime help seeking. Studies had to include results on the factors influencing help-seeking behaviour (IC 7).

We included studies if they fulfilled all of the inclusion criteria. If there were disagreements about the in- or exclusion of a study, the decision was discussed until consensus was reached (JLM, TS, ALB).

### Data extraction and synthesis

The study characteristics, factors associated with help seeking, results and methodological quality were extracted by JLM and TS. Qualitative data synthesis was performed to illustrate which influencing factors were investigated and to discuss heterogeneous findings (e.g., adjusted and unadjusted results) from samples in heterogeneous contexts (e.g., countries, health care systems). Therefore, JLM and TS classified all investigated variables into individual and contextual predisposing, enabling and need factors according to the ‘Behavioral Model of Health Service Use’ [15]. Data synthesis was performed by vote counting because of the heterogeneity of settings, measures, adjustments and the number of investigated variables. Therefore, measures (e.g., odds ratios, chi-square, and regression coefficients) of the association between each variable and help seeking were grouped into significant positive, significant negative and non-significant results and were listed for each variable. Any disagreements between JLM and TS were discussed until agreement was reached. We documented if and which potential confounding variables were adjusted for in the analyses.

### Assessment of methodological quality

Two researchers (JLM, TS) evaluated the methodological quality of all of the included studies. Because of the high level of homogeneity in study design, we considered only criteria with variance between studies. Consequently, three criteria were used (S4 Appendix). Two criteria of 14 from the Quality Assessment Tool for Observational Cohort and Cross-Sectional Studies [29] were selected to examine internal validity (Q1 and Q2, S4 Appendix). We added one criterion about the recruitment of a cohort from the Critical Appraisal Skills Programme [30] to focus on external validity (Q3, S4 Appendix). A score of 1 was awarded for each criterion adequately fulfilled, with a potential score ranging from 0 (poor) to 3 (excellent). No studies were excluded because of poor quality rating.

## Results

### Study characteristics

**Fig 2:**
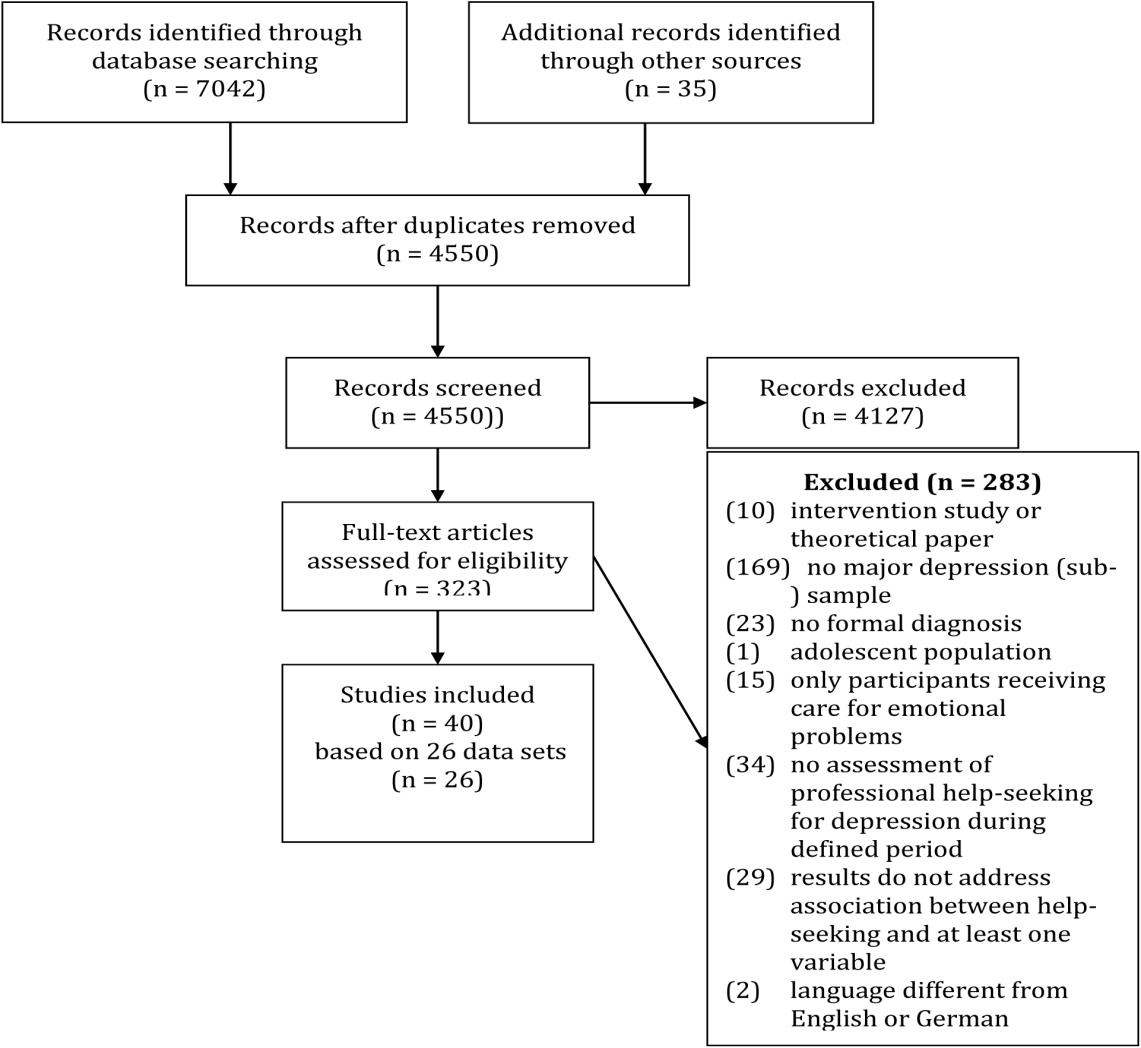
Systematic literature review flow diagram.

Altogether, 40 studies based on 26 datasets were included in the systematic review (see Fig 2 for an overview of the search process). The study characteristics are summarized in S5 Appendix. The 26 included datasets comprised 24 cross-sectional studies, one case-control study [31] and one cohort study [32]. The years of publication for these studies ranged from 1987 [33] to 2016 [34]. In 24 of the 26 datasets, the help-seeking behaviour of individuals with major depression was assessed in population-based samples within a certain region or country. The exceptions included a study investigating white-collar professionals from a specific corporation [35] and a study investigating the relatives and spouses of people seeking treatment for mental disorders and matched controls [31]. Most datasets were collected in the US (N = 10) and Canada (N = 8). The other datasets were collected in Finland (N = 3), Ethiopia (N = 1), Mexico (N = 1), Estonia (N = 1), Netherlands (N = 1) and Europe (N = 1). The sample sizes ranged between 102 and 18,927 participants with major depression [36].

### Quality of studies

The study quality was rated as ‘good’ (60%) for more than half of the studies, ‘excellent’ for one study [32], ‘fair’ for 35% of the studies, and ‘poor’ for one study [35]. S5 Appendix displays the ratings of methodological quality.

### Individual predisposing factors

Thirty-nine studies and all 26 datasets reported results on individual characteristics, as shown in S6 Appendix.

### Beliefs

Five studies assessed stigma and help-seeking beliefs; feeling comfortable with seeking help [37, 38] and having the intention to seek help [38] were positively associated with help-seeking behaviour. Negative attitudes towards antidepressants were negatively related to help-seeking behaviour [39]. Lin and Parikh [38] reported no significant associations between help seeking and beliefs about improving through or without professional care and feeling embarrassed about seeking help. This finding is inconsistent with Diala, Muntaner [37], who found that participants who said they would be embarrassed if their friends knew they were getting mental health care were less likely to use it than others. Aromaa, Tolvanen [39] found no association between help-seeking behaviour and prejudices against mentally ill people. However, they found that a stronger desire for social distance was negatively related to help seeking. Boerema, Kleiboer [34] reported that own negative attitudes towards people with depression are negatively related to help-seeking, whereas participants’ beliefs about how other people think about depression was unrelated to help-seeking.

Kleinberg, Aluoja [19] found that a higher external locus of control was associated with increased help seeking.

### Demographic factors

The associations between gender and help seeking were analysed in 16 datasets. In three US samples [18, 32, 40] and one Finish sample [39], being female was positively related to help-seeking behaviour. Sussman, Robins [33] reported the same association only among white Americans, not among black Americans. An association between gender and help-seeking was not found in Spanish [41], Ethiopian [42], Canadian [38, 40, 43–45], American [31, 35], Finnish [46, 47], Netherlands [34] or Mexican [48] samples.

The association between age and help seeking was analysed in 17 different datasets and was mainly computed comparing different age groups. In eight datasets, age was significantly associated with help-seeking behaviour. Two of these studies reported a positive association between age in years and help seeking [31, 39]. In the other five datasets, being middle-aged was significantly related to higher help-seeking rates [32, 41, 49–51].

### Social factors

The associations between help-seeking behaviour and social factors are shown in S6 Appendix. Results were available for educational status (N = 11 datasets), ethnicity (N = 9 datasets), family and living situation (N = 15 datasets) and employment (N = 4 datasets)

The associations between help seeking and education in individuals with major depression were significantly positive or non-significant in the eleven datasets. For example, in three datasets, more years of education and a higher degree were positively associated with help-seeking behaviour after adjusting for clinical and socio-demographic variables [32, 42, 44, 52]. After adjusting for clinical and socio-demographic variables, this positive association remained significant only in the Canadian dataset [40] and the US dataset [53].

Differences between help seeking by ethnic group were assessed in four Canadian and five US datasets. Belonging to a different ethnic group was defined differently between the studies. Differences in help-seeking between different ethnic groups were reported in seven studies [18, 32, 33, 37, 40, 54, 55]. For example, black Americans [55], African Americans [18, 37], Mexican Americans [18], and ethnic minorities [40] had lower rates of seeking help compared to whites. No differences were reported between the help-seeking behaviours of people born in Canada and of Canadian migrants [44], except that lower rates of help-seeking were observed in a group of Chinese immigrants compared to a group of Canadians born in Canada [52, 54]. The results from the ‘National Survey of American Life’ (NSAL) showed that although African Americans reported higher rates of seeking help than Caribbean Blacks, this difference was only significant in a sample of adults with severe or very severe symptoms [56] and was not significant in a sample of adults with mild to moderate symptoms [56] or in a subsample of mothers [57]. Sussman, Robins [33] reported that blacks had lower odds of seeking help than whites only in people with less severe depression.

Eight [33, 34, 38, 40–42, 44, 52] out of 15 datasets found no association between help-seeking behaviour and marital status or living as married. In addition, no significant associations were reported for cohabitation [19, 47], household size [19] or pregnancy [58]. However, four studies showed that being married or living as married was negatively associated with help-seeking behaviour [17, 31, 35, 59]; in contrast, Chartrand, Robinson [32] found the opposite relationship. Gadalla [52] reported that single mothers with adult children had the lowest odds of seeking treatment in comparison to other women.

### Individual enabling characteristics

Financial aspects were addressed in ten datasets, focusing mainly on income or household wealth. In Spanish respondents from the ‘European Study of the Epidemiology of Mental Disorders’ (ESEMeD), the low to average income group was negatively related to help seeking compared to the highest income group [41]. Diala, Muntaner [37] found a similar association in respondents from the ‘National Comorbidity Survey’ (NCS). Conflicting results were found in male respondents from the CCHS 1.2, in which help-seeking was positively related to a lower adjusted household income [17]. All other studies reported non-significant results regarding this association [18, 31, 32, 38, 40, 42, 44, 52, 53]. In the ‘Collaborative Psychiatric Epidemiology Survey’ (CPES), health insurance coverage doubled the odds of any use of depression therapy in the past year [18], while in the American samples in the ‘Joint Canada/United States Survey on Health’, this association lost significance in the multivariate model [40].

Regarding the influence of organizational factors on help-seeking behaviour, findings on the availability, accessibility and acceptability of care were available from the CCHS 1.2 [17]. Additionally, findings on the influence of having a regular medical doctor were available in the ‘Joint Canada/United States Survey on Health’ [40]. Availability, including waiting times and help not available in the area, was positively related to help-seeking among female Canadian respondents, whereas accessibility and acceptability were not related to help-seeking [17].

Social support was addressed in three datasets. In the CCHS 1.2, social support and help seeking were positively related in women only [17, 52]. Although social support was not directly associated with help-seeking behaviour in the Estonian health survey, emotional loneliness was associated with increased help seeking among depressed persons with an external locus of control [19]. Dew, Bromet [35] found that receiving social support during the index episode was negatively related to help seeking, whereas receiving recommendations from others to seek professional help was positively related to help seeking.

### Individual need characteristics

Studies on the need factors influencing help-seeking behaviour often focused on the severity of depression (14 datasets), psychiatric comorbidity (11 datasets), duration of episode (9 datasets), subjective disability (5 datasets), number of depressive episodes (6 datasets), somatic comorbidity (6 datasets), and presence of certain depressive symptoms (7 datasets) (S6 Appendix). Illness and symptom based need factors were assessed through structured interviews or questionnaires and were defined as professional judgements about people’s mental health status and therefore can be allocated to evaluated need, according to the “Behavioral Model of Health Services Use” [15]. Specifically, severity of depression was positively related to help-seeking in seven of the 16 datasets [31, 39, 40, 46, 47, 60, 61]. In addition, a longer duration of illness was positively related to help-seeking behaviour in six datasets [31, 34, 35, 40, 46] and was non-significantly related in three datasets [33, 38, 53]. After adjusting for socio-demographic and clinical variables, having more than one major depressive episode was no longer significantly associated with help seeking in the ‘Ontario Health Study’ (OHS) [38, 53]. Furthermore, in three other datasets, no significant association occurred [31, 35, 53]. However, in the group of black US participants [33] and female Canadians [52], there was a significant positive association. Having trouble concentrating [31, 35, 46] and suicidal thoughts or ideation [31, 35, 46, 52] were positively related to help-seeking behaviour. Conversely, three studies found no significant results for the latter association [32, 38, 53].

Psychiatric comorbidity was assessed in eleven datasets, and somatic comorbidity in seven (S6 Appendix). Having comorbid generalized anxiety disorder [17, 44, 47] or a panic disorder [31, 62] was positively related to help-seeking behaviour. Interestingly, after adjusting for several clinical and socio-demographic factors, having a generalized anxiety disorder, agoraphobia or panic disorder in the previous 12 months was significantly related to higher help-seeking rates in OHS respondents but not in NCS respondents [53]. In contrast with the findings from the Ontario study, Lin and Parikh [38] found no significant differences analysing the same dataset. Moreover, comorbid phobic disorders were not related to help-seeking behaviour [31].

Chen, Crum [36] showed that people suffering from major depression and substance dependence were more likely to seek help than people suffering from major depression only. Other findings indicate no significant difference in help-seeking behaviour with comorbid substance dependence disorder [17, 38, 44, 63] or alcohol or drug abuse [31]. Having any additional mental disorder was positively related to help-seeking behaviour in one [41] of four relevant studies [38, 44, 64].

Suffering from chronic somatic disorders was significantly associated with higher help-seeking rates in two datasets [17, 44, 52, 65]. However, in five datasets, this association was non-significant [34, 38, 40, 41, 60]. Demyttenaere, Bonnewyn [65] found that people with depression who had comorbid painful physical symptoms had lower rates of help seeking than those without these comorbid symptoms. In older people from the same dataset, this association was not significant [66].

### Contextual characteristics

Studies on the contextual characteristics of help seeking in individuals with major depression have focused on region or different countries. Living in an urban or rural area was not related to help-seeking behaviour in Spanish [41], Ethiopian [42], Canadian [38, 44] or American [32] samples. Additionally, no differences in help-seeking behaviour were found between the American and Canadian samples [40] or between the Francophone Canadian and European samples [67]. Differences in individuals’ help-seeking behaviour between different regions of the US were found in one [32] of two studies [31].

## Discussion

### Summary

This paper aimed to systematically review the individual and contextual characteristics associated with professional help-seeking behaviour in adults suffering from major depression based on the ‘Behavioral Model of Health Service Use’. Several studies investigated the association between help-seeking behaviour and individual characteristics, such as socio-demographic predisposing factors (e.g., age, gender, ethnicity, education, and family status), enabling factors (financial situation/income) and need factors (e.g., severity of depression, comorbidity, and duration and number of episodes). Some studies focused on beliefs (n = 4) (predisposing factors), social support (n = 4), organization (n = 3) (enabling factors), and context (n = 8) (e.g., urban vs. rural, country) and help-seeking behaviour. No study focusing on need for mental health treatment was included. Similarly, studies investigating help-seeking behaviour for different diseases based on the ‘Behavioral Model of Health Services Use’ examined characteristics similar to those of the studies included in our review [16].

Based on the current review, it appears that several factors may influence the likelihood that an individual suffering from major depression will seek professional help.

Predisposing factors that seem most likely to decrease help-seeking behaviour in individuals with major depression are, being young or elderly, being male, belonging to certain ethnic groups and having a lower educational status. Although these groups may be at a higher risk for not seeking professional help for major depression, the reasons for this higher risk need to be clarified. Certain structural or attitude-related barriers to seeking care among individuals in these groups may explain the findings. For instance, synthesizing qualitative studies, Doblyte and Jiménez-Mejías [24] identified attitudinal barriers for help seeking among depressed man, ethnic minorities and young adults: They concluded that help seeking is a threat to hegemonic masculinity, that the fear of disclosure and being judged was strongest among young adults and that ethnic minorities were more willing to keep depression within family [24]. Apart from attitudinal barriers, structural barrier like cultural inappropriateness of interventions could explain lower help-seeking rates among ethnic minorities [24].

The majority of studies reported no association between income and help-seeking behaviour. A possible explanation for this finding might be that income as an indicator is not sensitive enough to detect socioeconomic differences in the use of health care services [68]. Regardless, accounting for the financing of health care systems it is necessary to interpret these associations [15].

There is some evidence that the severity of depression, longer and more depressive episodes and the presence of anxiety disorders are related to higher help-seeking rates. These findings are consistent with those on help-seeking behaviour in individuals with depressive symptoms or depressive disorder [20]. However, as these findings were mainly based on retrospective cross-sectional studies, it remains unclear whether individuals affected by more severe depression are more likely to seek help. It is possible that individuals receiving treatment perceive their condition to be more severe than individuals without treatment. Qualitative findings indicate that the first hypothesis is more likely, because professional help-seeking is seen as the “final step”, because it “damages one’s self-definition” [24].

Based on the reviewed literature, the effects of additional individual predisposing factors such as attitudes on help-seeking behaviour and enabling factors like social support remain unclear. These psychosocial variables are mentioned in the ‘Behavioral Model of Health Service Use’, but which factors influence help-seeking behaviour in what way is not specified. Nonetheless, the initial findings show that social support might be associated with help-seeking behaviour [17, 35, 52]. Therefore, it might be worth distinguishing between informational social support (e.g., recommending seeking care) and emotional social support and investigating the interactions with other psychological concepts such as locus of control. Although the former could facilitate help seeking (e.g. [35]), the latter may only be associated with help seeking in certain populations (e.g., in individuals with an external locus of control [e. g. 19]). Regarding the influence of beliefs, feeling comfortable seeking care [37, 38] was positively associated with help-seeking, whereas having negative beliefs about antidepressants and having a stronger desire for social distance from people who are mentally ill [39] and having negative attitudes towards them [34] might have a negative impact on help-seeking behaviour. Within the ‘Health Beliefs Model’ [11], these beliefs could be considered the perceived benefits and barriers to taking action. Henshaw and Freedman‐Doan [69] conceptualised help-seeking for mental illnesses using this model and examined the role of fears about treatment and stigma as psychological barriers. The desire for social distance from mentally ill people is known to be an indirect measure of stigmatizing beliefs towards people belonging to this group, and a dissonance between these negative stereotypes and the preferred self can impede help-seeking for mental health problems [23]. Fears about antidepressant treatment could be a particular problem if practical or psychological barriers to seeking psychotherapy exist.

As evidenced by the findings presented in the results section, several factors of the ‘Behavioral Model of Health Service Use’ seem to be not validated through the systematic review. For instance, mainly no associations between certain predisposing factors (e.g. employment status), enabling factors (e.g. income, organisation), need factors (e.g. somatic symptoms, general health) and help-seeking were identified.

### Practical implications

The studies included in this review revealed that men, young and elderly adults, and people of certain ethnicities as well as individuals with a lower educational status with major depression are at risk of not seeking help, and these populations could be addressed in individually tailored interventions to increase help-seeking. In a review of randomized controlled trials, the majority of help-seeking interventions for depression, anxiety and psychological distress targeted young people [28]. In that review, Gulliver, Griffiths [28] provided some evidence that mental health literacy interventions (e.g., delivering destigmatisation information and/or providing information about help-seeking sources) can be effective in improving help-seeking attitudes. Mental health literacy is defined as “knowledge and beliefs about mental disorders which aid their recognition, management or prevention” [70]. However, this positive association could not confirmed for help-seeking behaviour for these interventions [28]. According to Doblyte and Jiménez-Mejías [24] who stressed out the role of hegemonic masculine identity and its influence in limiting men’s help seeking behaviour, educational campaigns for primary care providers can facilitate communication between male patients and GPs. Additionally a slighter entrance into care can be achieved. In this spirit, trainings which increase GPs intercultural competence and awareness of cultural differences regarding e.g. illness definition should also be considered [24]. However, further research on interventions that increase help-seeking intentions and behaviour among individuals suffering from major depression is needed.

### Limitations

The results of this review should be considered in light of several limitations. First, the vast majority of the studies reviewed were conducted in the US and Canada, which reduces the external validity of the findings. Second, the synthesis of results was limited because of the heterogeneity of the studies. Although the samples were homogenous regarding the formal diagnosis of major depression, the studies differed in terms of the samples’ age, gender and ethnicity as well as the health care systems affecting the participants. According to the ‘Behavioral Model of Health Service Use’, these contextual characteristics directly influence service utilization and indirectly influence service utilization through individual characteristics [15]. In addition, the results included different levels of adjustment. Third, reliable conclusions concerning whether a factor causes help-seeking behaviour were not possible, because the large majority of the studies used cross-sectional designs and retrospective data. Fourth, there was a lack of studies that quantitatively investigated the influence of individuals’ beliefs and perceptions on their help-seeking behaviour. Finally, because of the heterogeneous measures and adjustment methods used, a quantitative synthesis was not appropriate.

### Plea for consideration of the subjective perspective in help-seeking behaviour

The focus on socio-demographic and clinical variables in the reviewed literature is understandable, as the majority of the studies utilized secondary datasets, thus limiting the variables available for analysis. Nevertheless, it is important to obtain information on the subjective perspective to better understand the complex process of help seeking. Furthermore, including this perspective could provide insight into the associations between certain socio-demographic variables and help seeking. For instance, several studies have already been conducted to shed light on depressed men’s lower help-seeking rates (for review see [21]) and on men’s delays in medical and psychological help-seeking (for review see [71]). Specifically, embarrassment, distress or anxiety related to using health care services, need for emotional control, the perception of symptoms as minor and poor communication with health professionals were identified as barriers for help-seeking among men [71]. Although the ‘Behavioral Model of Health Service Use’ [15] does not focus on this subjective perspective, it is explicitly included in the predisposing contextual individual *beliefs* and implicitly included in *perceived need*. Psychological models such as the Self-Regulation Model of Illness Behavior [10], the Health Belief Model [11] and the Theory of Planned Behavior [12] focus on the individual’s perspective in the help-seeking process. According to these models, illness beliefs [10], perceived susceptibility and severity of symptoms as well as perceived expectations regarding treatment and self-efficacy [11, 12] and motivational factors [12] influence help-seeking behaviour. For instance, a qualitative analysis using the Self-Regulation Model found that primary care patients with depression who did not seek treatment believed that the treatment would not be effective, that depression would be short-lived and that it would not affect their daily lives [72]. Accordingly, it is promising to focus on psychological variables that affect the decision-making process of seeking help to better predict behaviour.

### Future directions for research

We suggest that future quantitative research on help-seeking behaviour among individuals suffering from major depression should focus more on the individuals’ perspective and include psychological theories as a framework for understanding the help-seeking process. Additionally, the influence of illness beliefs, treatment beliefs, anticipated stigmatization and perceived need for mental health care on help seeking may be worth investigating. Future research should provide insight into the associations between predisposing, enabling and need factors to improve the understanding of the complex process of help seeking. Therefore, the characteristics identified in the literature should be further considered.

Future prospective cohort studies on the causal relations between predisposing, enabling and need factors and help-seeking behaviour among individuals suffering from major depression should also be conducted. Measuring predisposing beliefs, perceived barriers, clinical variables, and perceived need prior to assessing help-seeking behaviour is important because these characteristics can change due to treatment and over time.

## Conclusion

This review found that the associations of help-seeking behaviour with socio-demographic predisposing (e.g., age, gender, ethnicity, education, and family status), enabling (financial situation/income), need (e.g., severity of depression, comorbidity, and duration and number of episodes) and contextual factors were investigated in several studies. Gender, age, education, ethnicity, marital status, severity of depression, duration and number of depressive episodes, and comorbid anxiety disorders appeared to influence help-seeking behaviour. Further research investigating the influence of these characteristics on help-seeking behaviour by individuals suffering from major depression in prospective cohorts and research specifically focused on beliefs, social support, organizational factors and perceived need for treatment would address a significant gap in the literature. A better understanding of the process of help-seeking by individuals suffering from major depression and improved knowledge of the factors that influence this process are important for identifying groups at risk of failing to seek adequate professional help and for improving their access to depression care.

## Supporting information

S1 Appendix PRISMA checklist. (DOC)

S2 Appendix Search strategy. (DOCX)

S3 Appendix Inclusion criteria (IC). (DOCX)

S4 Appendix Quality characteristics. Q1 and Q2 from the ‘Quality Assessment Tool for Observational Cohort and Cross-Sectional Studies” [29] Q3 from the Critical Appraisal Skills Programme [73].
(DOCX)

S5 Appendix Summary of the main characteristics of the published articles. ws = whole sample; MDE = major depressive episode; MDD = major depressive disorder; NR = not reported; DIS = Diagnostic Interview Schedule; WHO-CIDI / CIDI = World Health Organization’s composite international diagnostic interview; SFMD = Short form for major depression; SF = Short form; UM = Short Form (University of Michigan) ESEMeD = European Study on the Epidemiology of Mental Disorders; CCHS = Canadian Community Health Study; NESARC = National Epidemiologic Survey on Alcohol and Related Conditions; CPES = Collaborative Psychiatric Epidemiology Survey; NSAL = National Survey of American Life; NCS = National Comorbidity Survey; NCS-R = National Comorbidity Survey–Replication; NLAAS = National Latino and Asian American Study; JUCSH = Joint Canada/US Survey of Health; NSDUH = National Study on Drug Use and Health; OHS = Ontario Health Study; ENHS = Ethiopian National Health Survey; NPHS = National Population Health Survey; ENHS = Ethiopian National Health Survey.
(DOCX)

S6 Appendix Summary of results of the systematic review. If adjusted and unadjusted results were reported in the same study for the same variable, only the adjusted results were listed in the table. + = significant positive association between characteristic and help-seeking behaviour;— = significant negative association between characteristic and help-seeking behaviour; Ø = no significant association between characteristic and help-seeking behaviour; x = significant differences between different groups; ESEMeD = European Study of the Epidemiology of Mental Disorders; CCHS = Canadian Community Health Survey on Mental Health and Well Being; NESARC = National Epidemiologic Survey on Alcohol and Related Conditions; NSDUH = National Survey on Drug Use and Health; NCS = National Comorbidity Survey; OHS = Ontario Health Study; EHS = Estonian Health Survey; CPES = Collaborative Psychiatric Epidemiology Survey.
(DOCX)
