# Supplementary material for: Factors associated with help-seeking behaviour among individuals with major depression: A systematic review
Source: PLoS One. 2017 May 11;12(5):e0176730. doi: 10.1371/journal.pone.0176730 (PMC5426609; doi:10.1371/journal.pone.0176730)
Supplement: S2 Appendix — (DOCX) [file pone.0176730.s002.docx]

S2 Appendix

*Search Strategy*

| Database | Used search Syntax | |
| --- | --- | --- |
|  | Depression | Help-seeking |
| all databases  (keywords) | (depression or ‘depressive disorder’ or ‘affective disorder’ or dysthymi* or ‘mood disorder’).ti. | help-seek* or (seek* adj2 help) or (seek* adj2 treatment) or seek* behavior or seek* behavior or (seek* adj2 health service*) or health seek*).mp. OR  (‘service use’ or ‘service usage’ or ‘service utili*’ or ‘care utili*’ or ‘health utili*’ or ‘use of help’ or ‘use of adj2 service’ or ‘treatment utili*’ or ‘barriers to adj2 care’).mp. |
| Psycinfo  (standard vocabulary) | exp Major Depression/ | exp health care seeking behavior/ exp help seeking behavior/  exp Health Care Utilization/ exp Treatment Barriers/ |
| Medline  (standard vocabulary) | Depressive disorder/  Depression/ | - |
| Embase  (standard vocabulary) | Depression/ | exp Help Seeking Behavior/  exp Health Care Utilization/ |
