# Supplementary material for: Factors associated with help-seeking behaviour among individuals with major depression: A systematic review
Source: PLoS One. 2017 May 11;12(5):e0176730. doi: 10.1371/journal.pone.0176730 (PMC5426609; doi:10.1371/journal.pone.0176730)
Supplement: S3 Appendix — (DOCX) [file pone.0176730.s003.docx]

S3 Appendix

*Inclusion Criteria (IC)*

| Study design | |
| --- | --- |
| IC 1 | Is the design of the study a cross-sectional study or cross-sectional analysis at baseline or case-control study or a cohort study? |
| Population | |
| IC 2 | Is the study population or a subsample made of individuals with a major depressive episode or major depression disorder? |
| IC 3 | Was a reliable and valid diagnostic in accordance with DSM-III, DSM-III-R, DSM-IV, DSM-IV-R, ICD-9, ICD-10 or RDC performed? |
| IC 4 | Are mainly adults included in the study or a subsample? |
| IC 5 | Does the study population include individuals who do not receive care for emotional problems (population-based datasets)? |
| Outcome | |
| IC 6 | Is professional psychosocial help-seeking for depression or mental health or emotional problems during a defined period (e.g. 12 month) assessed? |
| IC 7 | Is the association between at least one factor and help-seeking examined in one of the possible ways   1. Comparison of continuous variable between individuals seeking help vs. individuals not seeking help (e.g. t-test, ANOVA) 2. Comparison of nominal variables between individuals seeking help vs. individuals not seeking help (e.g. Chi², proportions, Odds Ratio) 3. Logistic regression with help-seeking as the dependent variable |
