# Supplementary material for: Factors associated with help-seeking behaviour among individuals with major depression: A systematic review
Source: PLoS One. 2017 May 11;12(5):e0176730. doi: 10.1371/journal.pone.0176730 (PMC5426609; doi:10.1371/journal.pone.0176730)
Supplement: S4 Appendix — Q1 and Q2 from the ‘Quality Assessment Tool for Observational Cohort and Cross-Sectional Studies” [29] Q3 from the Critical Appraisal Skills Programme [73]. (DOCX) [file pone.0176730.s004.docx]

S4 Appendix

*Quality Characteristics*

| Quality Assessment | |
| --- | --- |
| Q1  Q2  Q3 | For the analyses in this paper, were the exposure(s) of interest measured prior to the outcome(s) being measured?  Were key potential confounding variables measured and adjusted statistically for their impact on the relationship between exposure(s) and outcome(s)?  Was the cohort recruited in an acceptable way?  (Was the cohort representative of a defined population? Was there something special about the cohort? Was everybody included who should have been included?) |
| Q1 and Q2 from the ‘Quality Assessment Tool for Observational Cohort and Cross-Sectional Studies” ([23](#_ENREF_23)) Q3 from the Critical Appraisal Skills Programme ([66](#_ENREF_66)). | |
