# Supplementary material for: Factors associated with help-seeking behaviour among individuals with major depression: A systematic review
Source: PLoS One. 2017 May 11;12(5):e0176730. doi: 10.1371/journal.pone.0176730 (PMC5426609; doi:10.1371/journal.pone.0176730)
Supplement: S5 Appendix — ws = whole sample; MDE = major depressive episode; MDD = major depressive disorder; NR = not reported; DIS = Diagnostic Interview Schedule; WHO-CIDI / CIDI = World Health Organization’s composite international diagnostic interview; SFMD = Short form for major depression; SF = Short form; UM = Short Form (University of Michigan) ESEMeD = European Study on the Epidemiology of Mental Disorders; CCHS = Canadian Community Health Study; NESARC = National Epidemiologic Survey on Alcohol and Related Conditions; CPES = Collaborative Psychiatric Epidemiology Survey; NSAL = National Survey of American Life; NCS = National Comorbidity Survey; NCS-R = National Comorbidity Survey–Replication; NLAAS = National Latino and Asian American Study; JUCSH = Joint Canada/US Survey of Health; NSDUH = National Study on Drug Use and Health; OHS = Ontario Health Study; ENHS = Ethiopian National Health Survey; NPHS = National Population Health Survey; ENHS = Ethiopian National Health Survey. (DOCX) [file pone.0176730.s005.docx]

S5 Appendix

*Summary of the main characteristics of the published articles*

| Author (Year) | Location | Design | Sampling  (response rate) | Sample | Diagnostic Instrument (criteria) | Help-seeking | Help-seeking period | Sample size (MD) | % help-seeking for mental health (MD) | % Female | Age range (mean, SD) | Quality-rating |
| --- | --- | --- | --- | --- | --- | --- | --- | --- | --- | --- | --- | --- |
| Demyttenaere et al. (2006) | Belgium, France, Germany, Netherlands, Spain | cross-sectional (ESEMeD; 2001-03) | Stratified, multistage, clustered-area, probability sample without replacement (61.2 %) | MDE  (12 month) in representative random sample of non-institutionalized adults | WHO-CIDI 3.0  (DSM-IV) | use of any service | 12 month | 857 | 32.8 (without painful physical symptoms)  44.1 (with painful physical symptoms) | 52.2 (ws) | ≥ 18 | good |
| Gabilondo et al. (2011) | Spain | cross-sectional (ESEMeD; 2001-03) | Stratified, multistage, clustered-area, probability sample without replacement (78.6 %) | MDE  (12 month) in representative random sample of non-institutionalized adults | WHO-CIDI 3.0  (DSM-IV) | use of any service | 12 month | 247 | 59.1 | 74.1 | ≥ 18 | fair |
| Bonnewyn et al. (2009) | Belgium, France, Germany, Netherlands, Spain | cross-sectional (ESEMeD; 2001-03) | Stratified, multistage, clustered-area, probability sample without replacement (61.2 %) | MDE  (12 month) in representative random sample of non-institutionalized adults | WHO-CIDI 3.0  (DSM-IV) | use of any service | 12 month | 128 | 29.3 | 59.0 (ws) | ≥ 65 | fair |
| Tempier et al. (2010) | Canada, France, Belgium | cross-sectional (ESEMeD; 2001-03 & CCHS 1.2; 2002) | stratified, multistage, clustered-area, probability sample without replacement (77 %: Canada, 51 %: Belgium, 46 %: France (ws)) | MDE  (12 month), in representative sample of adult Francophones without anxiety disorder | WHO-CIDI 3.0  (DSM-IV) | use of any service | 12 month | 729 | 44.6 (European) 45.8 (Canadian) | 51.1  (Canadian) 52.2 (European) (ws) | ≥ 18 | fair |
| Hailemariam et al. (2012) | Ethiopia | cross-sectional (ENHS; 2003) | Stratified, multistage sample survey, selected from households (98,7% (ws)) | MDE  (12 month) in representative sample of adults | CIDI  (DRC) | Use of any health service | 12 month | 449 | 22.9 | 53.9 | ≥ 18  (36.1; 15.4) (ws) | good |
| Bland et al. (1997) | Canada (Edmonton) | cross-sectional national household survey (1986-1992) | Systematic random sample (86 %) of a national household sample (73%) (ws) | MDE  (12 month) in representative sample of adults | DIS  (DSM-III) | seen a care professional | 12 month | 171 | 37.6 (no comorbidity)  50.1 (comorbidity) | NR | ≥ 18 | fair |
| Tiwari & Wang (2008) | Canada | cross-sectional (CCHS-1.1; 2003) | two-stage stratified random sampling (households, over-representation of young persons and seniors) | MDE  (12 month) in representative sample of non-institutionalized inhabitants | CIDI-SFMD (DSM-III-R) | seen or talked to a health professional | 12 month | 9071* | 26.9 (Southeast Asian)  46.2  (white) | 47.8 (Chinese) 53.5 (Southeast Asian) (ws) | ≥ 12 | good |
| Crabb & Hunsley (2006) | Canada | cross-sectional (CCHS-1.1; 2000-2001) | 3 sampling frames (area frame, a random digit dialing, list frame) | MD  (12 month) in representative sample of non-institutionalized inhabitants | CIDI-SFMD (DSM-III) | seen or talked to a health professional | 12 month | 3550 | NR | NR | ≥ 45 | fair |
| Smith et al. (2013) | Canada (Ontario) | cross-sectional (CCHS-1.1; 2000-2001) | 3 sampling frames (area frame, a random digit dialing, list frame) | MD  (12 month) in representative sample of non-institutionalized inhabitants | CIDI-SFMD (DSM-III) | at least one visit to a physician | 12 month | 2085 | 44.7 | 70.3 | 18-74 | fair |
| Gadalla (2008) | Canada | cross-sectional (CCHS-1.2; 2002) | randomly selected using a stratified, multistage, clustered area sample | MDE  (12 month), in representative sample of non-institutionalized women | WMH-CIDI (DSM-IV) | contact with health care providers | 12 month | 1186 | 56.4 | 100 | ≥ 15 | good |
| Gagne et al. (2014) | Canada | cross-sectional (CCHS-1.2; 2002) | randomly selected using a stratified, multistage, clustered area sample | MDE  (12 month) in nationally representative sample of non-institutionalized individuals | WMH-CIDI (DSM-IV) | outpatient contact with different health care providers | 12 month | 1743 | 54.3 | 62.2 | ≥ 15 | good |
| Wang et al. (2005) | Canada | cross-sectional (CCHS-1.2; 2002) | randomly selected using a stratified, multistage, clustered area sample | MDD  (12 month) without lifetime mania in nationally representative sample of non-institutionalized individuals | WMH-CIDI (DSM-IV) | contact with health care providers | 12 month | 1563 | 59 | NR | ≥ 15 | good |
| Cheung et al. (2007) | Canada | cross-sectional (CCHS-1.2; 2002) | randomly selected using a stratified, multistage, clustered area sample | MDD  (12 month) in  nationally representative sample of non-institutionalized individuals | WMH-CIDI (DSM-IV) | contact with help service providers | 12 month | NR | 52.5 (man)  55.6 (women) | NR | 19-24 | fair |
| Manetti et al. (2014) | US, Hawaii, Alaska | cross-sectional  NESARC (2001-2002) | 18-24 year olds, Non-Hispanic Blacks and Hispanic respondents where oversampled | MDD  (12 month) in nationally representative community sample of US, Hawaii and Alaska civilian, non-institutionalized participants | AUDADIS-IV  (DSM-IV) | mental health specialist, hospital, emergency room or medication | 12 month | 3119 | 24.2 (≥ 65 years)  28.8 (< 65 years old) | 66.6 (< 65 years old) 71.6 (≥ 65 years old) | ≥ 18  (39.5; 0.1: < 65 years old) (74.68; 0.1: ≥ 65 years old) | good |
| Chartrand et al. (2012) | US, Hawaii, Alaska | cohort study  NESARC  Wave 1 (2001-2002) and Wave 2 (2004-2005) | 18-24 year olds, Non-Hispanic Blacks and Hispanic respondents where oversampled | MDD  (12 month) at Wave 1 in nationally representative community sample of US, Hawaii and Alaska civilian, non-institutionalized participants | AUDADIS-IV  (DSM-IV) | mental health specialist, hospital, emergency room or medication | 12 month + between Wave 1 and 2 | 2864 | 44.5 (past year at Wave 1)  29.5 (treatment utilization at Wave 2) | NR | ≥ 18 | excellent |
| Mackenzie et al. (2012) | US, Hawaii, Alaska | cross-sectional  NESARC, Wave 2 | 18-24 year olds, Non-Hispanic Blacks and Hispanic respondents where oversampled | MDD  (12 month) in nationally representative community sample of US, Hawaii and Alaska civilian, non-institutionalized participants (Wave 2: 34653) | AUDADIS-IV  (DSM-IV) | mental health specialist, hospital, emergency room or medication | 12 month | NR | 37.8 | NR | 20 - 64 | good |
| Hankerson et al. (2011) | US, Hawaii, Alaska | cross-sectional  NESARC (2001-2002) | 18-24 year olds, Non-Hispanic Blacks and Hispanic respondents where oversampled | MDD  (12 month) in nationally representative community sample of US, Hawaii and Alaska civilian, non-institutionalized participants | AUDADIS-IV  (DSM-IV) | mental health specialist, hospital, emergency room or medication | 12 month | 1866 | 48.3 –  67.3 | NR | ≥ 18 | good |
| Mojtabai & Olfson (2006) | US, Canada | cross-sectional  2002–2003 Joint Canada/United States Survey of Health | household selection (random digit dialing), random selection of members, oversampling: older than 65, excluded Canadian and US territories | MDE  (12 month) in representative community-dwelling non-institutionalized adults | CIDI-SF (DSM-IV) | seen or talked on the telephone to a health professional | 12 month | 447 (US)  304 (Canada) | 52 (USA)  56 (Canada) | 62.9 (US)  61.1 (Canada) | ≥ 18 | good |
| Ko et al. (2012) | US (Columbia) | cross-sectional  NSDUH (2005-2009) | stratified, multistage, clustered-area, probability sample | MDE  (12-month) in civilian, non-institutionalized, women | 9 questions (DSM-IV) | receiving prescription medication, counseling or inpatient care | 12 month | 375 (pregnant) + 8657 (not pregnant) | 46.9 (pregnant) 53.7 (not pregnant) | 100 | 18-44 | good |
| Chen et al. (2013) | US (Columbia) | cross-sectional  NSDUH (2005-2010) | stratified, multistage, clustered-area, probability sample | MDE  (12-month) in civilian, non-institutionalized population | CIDI (DSM-IV) | mental health treatment (outpatient, inpatient, medication) | 12 month | 18972 | 53.4 (no substance dependence) 60.5 (with non-alcohol drug dependence) | NR | ≥ 18 | good |
| Diala et al. (2000) | US | cross-sectional  NCS (1990-1992) | household selection at random, weighted sample to approximate the U.S population for age, sex, ethnicity, education | MDE  (12-month) in nationally representative household survey of non-institutionalized individuals | CIDI (DSM-III-R) | any visits to a psychologist or psychiatrist | 12 month | 504 | NR | 55.4 (white)  76.8 (African American) | 15-54 | good |
| Roy-Byrne et al. (2000) | US | cross-sectional  NCS (1990-1992) | household selection at random, weighted sample to approximate the U.S population for age, sex, ethnicity, education | MDE  (12 month) in nationally representative household survey of non-institutionalized individuals | CIDI (DSM-III-R) | any medical service (general medical, mental health), social services and self-help groups | 12 month | 588 | 35.5 (no Panic disorder)  55.2 (Panic disorder) | NR | 15-54 | good |
| Katz et al. (1998) | US, Ontario | cross-sectional  NCS (1990-1992) + OHS |  | MDE  (12 month) in nationally representative household survey of non-institutionalized individuals | modified CIDI (DSM-III-R) | any contact with a general medical or specialty provider | 12 month | 824 | 30.4 (US) 57.7 (Ontario) | 64.9 (US)  68.1 (Ontario) | 21-54 (35.6; 5: US) (36.2; 9: Ontario) | good |
| Lin & Parikh (1999) | Canada | cross-sectional  OHS (1990-1991) | Ontario household selection using a stratified, multi-stage sampling design | MDE  (12 month) in representative community sample of civilians | UM-CIDI (University of Michigan) (DSM-II-R) | professional inpatient or outpatient contacts | 12 month | 341 | 50.1 | NR | 15-64 | good |
| Kleinberg et al. (2013) | Estonia | cross-sectional  Estonian Health Survey (2006) | stratified random systematic sampling | MDE  (current, past 2 weeks) in non-institutionalized individuals | MINI (DSM-IV, ICD-10) | sought help | 12 month | 343 | 34.1 | 65.6 | 18-84 | good |
| Kleinberg et al. (2013) | Estonia | cross-sectional  Estonian Health Survey (2006) | stratified random systematic sampling | MDE  (current, past 2 weeks) in non-institutionalized individuals | MINI (DSM-IV, ICD-10) | sought help | 12 month | 343 | 34.1 | 65.6 | 18-84 | good |
| Aromaa et al. (2011) | Finland | cross-sectional  Finish population survey | randomly selected from population register (10000) | MDD  (12 month) in nationally representative survey of individuals | CIDI-SF (DSM-IV) | any health services | 12 month | 558 | 39.6 | 68.0 | 15-80 (50.6; 17.3) (ws) | good |
| Coryell et al. (1995) | US | case-control study  National Institute of Mental Health Program on the Psychobiology of Depression - Clinical Studies | randomly selected subset of relatives from study participants with MD, mania or schizoaffective disorder, sex- and age matched comparison subjects | MDD  (at baseline) in first-degree relatives, current spouses of people seeking treatment for MD, mania or schizoaffective disorder and matched controls | Schedule for Affective Disorders and Schizophrenia (RDC) | psychotherapy, ECT, medication or were hospitalized for at least one episode | 6 years | 547 | 57.2 | 65.8 (no treatment) 70.3 (treatment) | ≥17 | good (clinical variables) |
| Dew et al. (1991) | US | cross-sectional  Westinghouse Electric Corporation 1987-1988 | multi-stage sample recruitment procedure in two units of an electric company  (Introductory letters, contact by a project interviewer, articles in newsletter) | MD  (12 month) in white collar professionals (primarily engineers) from Westinghouse Electric Corporation | SCID (DSM-III) | consulting a professional | 12 month | 186 | 31.7 | NR | NR | poor |
| Hamalainen et al. (2004) | Finland | cross-sectional  Health Care Survey 1996 (FINHCS '96) | one-stage cluster sampling in which households formed the clusters (random) | MDE  (12 month) in nationally representative household survey of non-institutionalized persons | UM-CIDI Short Form (University of Michigan) (DSM-III-R) | health service use | 12 month | 557 | 27 | 65.89 | 15–75 (43) | good |
| Hamalainen et al. (2008) | Finland | cross-sectional  Health 2000 study (2000 - 2001) | two-stage stratified cluster-sampling | MDE  (12 month) in representative sample of Finland's general adults aged 30 and older living in mainland Finland | M-CIDI (DSM-IV) | health services | 12 month | 298 | 30 (men)  36 (women) | 69 | ≥ 30 | good |
| Rafful et al. (2012) | Mexico | cross-sectional  Mexican National Comorbidity Survey | stratified, multistage area probability Mexican urban household sample | MDE  (12 month) in representative sample of persons in the non-institutionalized population living in urban areas of Mexico | WHO-CIDI (DSM-IV) | talked to or consulted a medical doctor or other professional | 12 month | 531 | 28.9 (women) 36.7 (men) | 77.02 | 18-65 | fair |
| Sussman et al. (1987) | US  (St. Louis) | cross-sectional  Epidemiologic Catchment Area project (1981-1982) | household sample, blacks were oversampled | MDE  (6 month) in  representative sample of individuals | DIS (DSM-III) | spoke to a professional (outpatient, inpatient) | 6 month (outpatient) 12 month (inpatient) | 116 | 47.3 (white)  49.3 (black) | 77.4 (black) 73.2 (white) | ≥ 18  (40.2: black), (40.1; white) | fair |
| Wang et al. (2004) | Canada | cross-sectional  NPHS (National Population Health Survey) (1996-97) | multistage, stratified random sampling procedure | MDE  (12 month) in nationally representative sample of individuals | CIDI-SF for Major Depression (DSM-IV) | psychiatrist or psychologist, antidepressants by general practitioners or family doctors | 12 month | 3133 | 47.3 (with alcohol dependence)  47.8 (without alcohol dependence) | 50.9  (ws) | ≥ 12 | fair |
| Birnbaum et al. (2010) | US | cross-sectional  NCS-Replication (new Sample: 2001-2002) | over-sampling those with clinically significant psychopathology, post stratified to approximate the distribution of the 2000 Census on a range of socio-demographic variables | MDD  (12 month) in nationally representative community survey of respondents who were in the US workforce (employed / unemployed and looking for a job / disabled) | CIDI (DSM-IV) | receiving treatment | 12 month | 539 | 56.3 | NR | ≥ 18 | fair |
| Gonzalez et al. (2010) | US | cross-sectional  CPES (NSAL, NCS-R, NLAAS) 2001-2003 | multistage area probability sampling methods (sampling frames, sample selection procedures) | MDE  (12 month) in three nationally representative samples non-institutionalized Mexican-American, Puerto Rican, Caribbean Black, African-American, Non-Latino white adults | CIDI (DSM-IV) | Pharmacotherapy and/or psychotherapy | 12 month | 1306 | 50.76 | 52.43 (ws) | ≥ 18 | good |
| Boyd et al. (2011) | US | cross-sectional  NSAL (2001-2003) | probability household sample (household) oversampled Caribbean Blacks | MDE  (12 month) in nationally representative sample of African American, non-Hispanic white and Caribbean Black mothers | CIDI (DSM-IV) | Help-seeking | 12 month | NR | 17.70 (Caribbean Blacks)  26.09 (African American) | 100 | ≥ 18 | fair |
| Williams et al. (2007) | US | cross-sectional  NSAL (2001-2003) | probability household sample (household) oversampled Caribbean Blacks | MDD  (12 month) in nationally representative sample of African American, non-Hispanic white and Caribbean Black adults | WHO-CIDI (DSM-IV) | saw any health provider | 12 month | 276 | 24.3 (Caribbean Blacks)  45.0 (African American) | 49.1 (Caribbean Blacks)  56.0 (African American) | ≥ 18 | fair |
| Cairney & Wade (2002) | Canada | cross-sectional  NPHS (National Population Health Survey) (1994-95) | multistage, stratified random sampling procedure | MDE  (12 month) in representative sample of mothers with at least one child under age of 25 | CIDI-SF (DSM-III-TR, ICD-10) | seen and talked to a health professional | 12 month | 238 | 58.4 | 100 | 15-54 | fair |
| Boerema et al. (2016) | Netherlands | cross-sectional | random sample of adult population (28%) | MDD  (6 month) in representative random sample of adults | CIDI 2.1 (DSM-IV) | at least one contact with a mental health care provider | 6 month | 102 | 65 | 54.0 | 22-88 (52) | good |

*Note:* ws = whole sample; MDE = major depressive episode; MDD = major depressive disorder; NR = not reported; DIS = Diagnostic Interview Schedule; WHO-CIDI / CIDI = World Health Organization’s composite international diagnostic interview; SFMD = Short form for major depression; SF = Short form; UM = Short Form (University of Michigan)

ESEMeD = European Study on the Epidemiology of Mental Disorders; CCHS = Canadian Community Health Study; NESARC = National Epidemiologic Survey on Alcohol and Related Conditions; CPES = Collaborative Psychiatric Epidemiology Survey; NSAL = National Survey of American Life; NCS = National Comorbidity Survey; NCS-R = National Comorbidity Survey – Replication; NLAAS = National Latino and Asian American Study; JUCSH = Joint Canada/US Survey of Health; NSDUH = National Study on Drug Use and Health; OHS = Ontario Health Study; ENHS = Ethiopian National Health Survey; NPHS = National Population Health Survey; ENHS = Ethiopian National Health Survey.
