# Supplementary material for: Factors associated with help-seeking behaviour among individuals with major depression: A systematic review
Source: PLoS One. 2017 May 11;12(5):e0176730. doi: 10.1371/journal.pone.0176730 (PMC5426609; doi:10.1371/journal.pone.0176730)
Supplement: S6 Appendix — If adjusted and unadjusted results were reported in the same study for the same variable, only the adjusted results were listed in the table. + = significant positive association between characteristic and help-seeking behaviour;— = significant negative association between characteristic and help-seeking behaviour; Ø = no significant association between characteristic and help-seeking behaviour; x = significant differences between different groups; ESEMeD = European Study of the Epidemiology of Mental Disorders; CCHS = Canadian Community Health Survey on Mental Health and Well Being; NESARC = National Epidemiologic Survey on Alcohol and Related Conditions; NSDUH = National Survey on Drug Use and Health; NCS = National Comorbidity Survey; OHS = Ontario Health Study; EHS = Estonian Health Survey; CPES = Collaborative Psychiatric Epidemiology Survey. (DOCX) [file pone.0176730.s006.docx]

S6 Appendix

*Summary of results of the systematic review*

|  | Positive findings | Neutral findings | Negative findings | Demyttenaere et al. (2006) | Gabilondo et al. (2011) | Bonnewyn et al. (2009) | Tempier et al. (2010) | Hailemariam et al. (2012) | Bland et al. (1997) | Tiwari & Wang (2008) | Crabb & Hunsley (2006) | Smith et al. (2013) | Gadalla (2008) | Gagne et al. (2014) | | Wang et al. (2005) | Cheung & Dewa (2007) | Manetti et al. (2014) | Chartrand et al. (2012) | Mackenzie et al. (2012) | Hankerson et al. (2011) | Mojtabai & Olfson (2006) | | Ko et al. (2012) | Chen et al. (2013) | Diala et al. (2000) | Roy-Byrne et al. (2000) | Katz et al. (1998) | | Lin & Parikh (1999) | Kleinberg, et al. (2013b) | Kleinberg, et al. (2013a) | Aromaa et al. (2011) | Coryell et al. (1995) | Dew et al. (1991) | Hamalainen et al. (2004) | Hamalainen et al. (2008) | Rafful, et al. (2012) | | Sussman et al. (1987) | | Wang & El-Guebaly (2004) | Birnbaum et al. (2010) | Gonzalez et al. (2010) | Boyd et al. (2011) | Williams et al. (2007) | Cairney & Wade (2002) | Boerema et al. (2016) |
| --- | --- | --- | --- | --- | --- | --- | --- | --- | --- | --- | --- | --- | --- | --- | --- | --- | --- | --- | --- | --- | --- | --- | --- | --- | --- | --- | --- | --- | --- | --- | --- | --- | --- | --- | --- | --- | --- | --- | --- | --- | --- | --- | --- | --- | --- | --- | --- | --- |
|  | + | 0 | - |  |  |  |  |  |  |  |  |  |  | ♀ | ♂ |  |  |  |  |  |  | U | C |  |  |  |  | U | C |  |  |  |  |  |  |  |  | ♀ | ♂ | B | W |  |  |  |  |  |  |  |
| Dataset |  |  |  | a | a | a | af | b | c | d | e | e | f | f | f | f | f | g | g | g | g | h | i | j | j | k | k | k | l | l | m | m | n | o | p | q | r | s | s | t | t | u | v | vwx | x | x | y | z |
| ***Predisposing*** |  |  |  |  |  |  |  |  |  |  |  |  |  |  |  |  |  |  |  |  |  |  |  |  |  |  |  |  |  |  |  |  |  |  |  |  |  |  |  |  |  |  |  |  |  |  |  |  |
| **Demographic** |  |  |  |  |  |  |  |  |  |  |  |  |  |  |  |  |  |  |  |  |  |  |  |  |  |  |  |  |  |  |  |  |  |  |  |  |  |  |  |  |  |  |  |  |  |  |  |  |
| Female gender | 5 | 13 | 0 |  | Ø |  |  | Ø |  |  |  | Ø |  |  |  | Ø | Ø |  | + |  |  | + | Ø |  |  |  |  |  |  | Ø |  |  | + | Ø | Ø | Ø | Ø | Ø | | Ø | + |  |  | + |  |  |  |  |
| Age |  | 13 |  |  | x |  |  | Ø |  |  | x |  | Ø | x | Ø | x |  | x | x | x |  | Ø | Ø |  |  |  |  |  |  |  |  | Ø | x | x | Ø | Ø | Ø | x | Ø | Ø | Ø |  |  | x |  |  |  | Ø |
| **Social** |  |  |  |  |  |  |  |  |  |  |  |  |  |  |  |  |  |  |  |  |  |  |  |  |  |  |  |  |  |  |  |  |  |  |  |  |  |  |  |  |  |  |  |  |  |  |  |  |
| Higher education | 7 | 8 | 0 |  | Ø |  |  | + |  |  |  |  | + | + | + | Ø |  |  | + |  |  | Ø | + |  |  |  |  | + | Ø | Ø |  |  |  | Ø | Ø |  |  |  |  |  |  |  |  | Ø |  |  |  |  |
| ‚ethnic group‘ |  | 4 |  |  |  |  |  |  |  | x |  |  | Ø |  |  |  |  |  | x |  | x | x | x |  |  | x |  |  |  | Ø |  |  |  |  |  |  |  |  |  | x | |  |  | x | Ø | Ø |  |  |
| employed | 0 | 3 | 1 |  | - |  |  | Ø |  |  |  |  |  |  |  |  |  |  |  |  |  |  |  |  |  |  |  |  |  | Ø |  | Ø |  |  |  |  |  |  |  |  | |  |  |  |  |  |  |  |
| (living as) married | 1 | 10 | 5 |  | Ø |  |  | Ø |  |  |  |  | Ø | - | - | Ø |  |  | + |  |  | Ø | Ø |  |  |  |  |  |  | Ø |  |  |  | - | - |  |  |  |  | Ø | Ø |  |  |  |  |  | - | Ø |
| Single-parent child ≥ 25 | 0 | 0 | 1 |  |  |  |  |  |  |  |  |  | - |  |  |  |  |  |  |  |  |  |  |  |  |  |  |  |  |  |  |  |  |  |  |  |  |  |  |  | |  |  |  |  |  |  |  |
| Single-parent child < 25 | 0 | 1 | 0 |  |  |  |  |  |  |  |  |  | Ø |  |  |  |  |  |  |  |  |  |  |  |  |  |  |  |  |  |  |  |  |  |  |  |  |  |  |  | |  |  |  |  |  |  |  |
| Cohabitation | 0 | 2 | 0 |  |  |  |  |  |  |  |  |  |  |  |  |  |  |  |  |  |  |  |  |  |  |  |  |  |  |  | Ø |  |  |  |  |  | Ø |  |  |  | |  |  |  |  |  |  |  |
| Household size | 0 | 1 | 0 |  |  |  |  |  |  |  |  |  |  |  |  |  |  |  |  |  |  |  |  |  |  |  |  |  |  |  | Ø |  |  |  |  |  |  |  |  |  | |  |  |  |  |  |  |  |
| Pregnancy | 0 | 1 | 0 |  |  |  |  |  |  |  |  |  |  |  |  |  |  |  |  |  |  |  |  | Ø |  |  |  |  |  |  |  |  |  |  |  |  |  |  |  |  | |  |  |  |  |  |  |  |
| Others recommended help | 1 | 0 | 0 |  |  |  |  |  |  |  |  |  |  |  |  |  |  |  |  |  |  |  |  |  |  |  |  |  |  |  |  |  |  |  | + |  |  |  |  |  | |  |  |  |  |  |  |  |
| Loneliness | 0 | 2 | 0 |  |  |  |  |  |  |  |  |  |  |  |  |  |  |  |  |  |  |  |  |  |  |  |  |  |  |  | Ø |  |  |  |  |  |  |  |  |  | |  |  |  |  |  |  | Ø |
| Satisfaction with couple relationship | 0 | 1 | 0 |  |  |  |  |  |  |  |  |  |  |  |  |  |  |  |  |  |  |  |  |  |  |  |  |  |  |  | Ø |  |  |  |  |  |  |  |  |  | |  |  |  |  |  |  |  |
| Membership in at least one organization | 0 | 1 | 0 |  |  |  |  |  |  |  |  |  |  |  |  |  |  |  |  |  |  |  |  |  |  |  |  |  |  |  | Ø |  |  |  |  |  |  |  |  |  | |  |  |  |  |  |  |  |
| Religion | 0 | 1 | 0 |  |  |  |  |  |  |  |  |  |  |  |  |  |  |  |  |  |  |  |  |  |  |  |  |  |  |  |  |  |  | Ø |  |  |  |  |  |  | |  |  |  |  |  |  |  |
| **Beliefs** |  |  |  |  |  |  |  |  |  |  |  |  |  |  |  |  |  |  |  |  |  |  |  |  |  |  |  |  |  |  |  |  |  |  |  |  |  |  |  |  | |  |  |  |  |  |  |  |
| Comfortable seeking care | 2 | 0 | 0 |  |  |  |  |  |  |  |  |  |  |  |  |  |  |  |  |  |  |  |  |  |  | + |  |  |  | + |  |  |  |  |  |  |  |  |  |  | |  |  |  |  |  |  |  |
| Positive treatment beliefs | 0 | 1 | 0 |  |  |  |  |  |  |  |  |  |  |  |  |  |  |  |  |  |  |  |  |  |  |  |  |  |  | Ø |  |  |  |  |  |  |  |  |  |  | |  |  |  |  |  |  |  |
| Negative antidepressant beliefs | 0 | 0 | 1 |  |  |  |  |  |  |  |  |  |  |  |  |  |  |  |  |  |  |  |  |  |  |  |  |  |  |  |  |  | - |  |  |  |  |  |  |  | |  |  |  |  |  |  |  |
| Definitely seek professional help if serious mental illness | 1 | 0 | 0 |  |  |  |  |  |  |  |  |  |  |  |  |  |  |  |  |  |  |  |  |  |  |  |  |  |  | + |  |  |  |  |  |  |  |  |  |  | |  |  |  |  |  |  |  |
| External locus of control | 1 | 0 | 0 |  |  |  |  |  |  |  |  |  |  |  |  |  |  |  |  |  |  |  |  |  |  |  |  |  |  |  | + |  |  |  |  |  |  |  |  |  | |  |  |  |  |  |  |  |
| Get better on own | 0 | 1 | 0 |  |  |  |  |  |  |  |  |  |  |  |  |  |  |  |  |  |  |  |  |  |  |  |  |  |  | Ø |  |  |  |  |  |  |  |  |  |  | |  |  |  |  |  |  |  |
| Embarrassed friend knew | 0 | 1 | 1 |  |  |  |  |  |  |  |  |  |  |  |  |  |  |  |  |  |  |  |  |  |  | - |  |  |  | Ø |  |  |  |  |  |  |  |  |  |  | |  |  |  |  |  |  |  |
| Desire for social distance | 0 | 0 | 1 |  |  |  |  |  |  |  |  |  |  |  |  |  |  |  |  |  |  |  |  |  |  |  |  |  |  |  |  |  | - |  |  |  |  |  |  |  | |  |  |  |  |  |  |  |
| Depression is a matter of will | 0 | 1 | 0 |  |  |  |  |  |  |  |  |  |  |  |  |  |  |  |  |  |  |  |  |  |  |  |  |  |  |  |  |  | Ø |  |  |  |  |  |  |  | |  |  |  |  |  |  |  |
| Perceived stigma | 0 | 1 | 0 |  |  |  |  |  |  |  |  |  |  |  |  |  |  |  |  |  |  |  |  |  |  |  |  |  |  |  |  |  |  |  |  |  |  |  |  |  | |  |  |  |  |  |  | Ø |
| Personal stigma | 0 | 0 | 1 |  |  |  |  |  |  |  |  |  |  |  |  |  |  |  |  |  |  |  |  |  |  |  |  |  |  |  |  |  |  |  |  |  |  |  |  |  | |  |  |  |  |  |  | - |
| **Other** |  |  |  |  |  |  |  |  |  |  |  |  |  |  |  |  |  |  |  |  |  |  |  |  |  |  |  |  |  |  |  |  |  |  |  |  |  |  |  |  | |  |  |  |  |  |  |  |
| neuroticism | 0 | 1 | 0 |  |  |  |  |  |  |  |  |  |  |  |  |  |  |  |  |  |  |  |  |  |  |  |  |  |  |  |  |  |  |  |  |  |  |  |  |  | |  |  |  |  |  |  | Ø |
| Relatives/spouses with affective illness | 0 | 3 | 0 |  |  |  |  |  |  |  |  |  |  |  |  |  |  |  |  |  |  |  |  |  |  |  |  |  |  | Ø |  |  |  | Ø | Ø |  |  |  |  |  | |  |  |  |  |  |  |  |
| Prior help-seeking behaviour | 2 | 0 | 0 |  |  |  |  |  |  |  |  |  |  |  |  |  |  |  |  |  |  |  |  |  |  |  |  |  |  | + |  |  |  |  | + |  |  |  |  |  | |  |  |  |  |  |  |  |
| Childhood abuse | 0 | 1 | 0 |  |  |  |  |  |  |  |  |  |  |  |  |  |  |  |  |  |  |  |  |  |  |  |  |  |  | Ø |  |  |  |  |  |  |  |  |  |  | |  |  |  |  |  |  |  |
| Low daily stress | 1 | 0 | 0 |  |  |  |  |  |  |  |  |  | + |  |  |  |  |  |  |  |  |  |  |  |  |  |  |  |  |  |  |  |  |  |  |  |  |  |  |  | |  |  |  |  |  |  |  |
| ***Enabling*** |  |  |  |  |  |  |  |  |  |  |  |  |  |  |  |  |  |  |  |  |  |  |  |  |  |  |  |  |  |  |  |  |  |  |  |  |  |  |  |  | |  |  |  |  |  |  |  |
| **Financing** |  |  |  |  |  |  |  |  |  |  |  |  |  |  |  |  |  |  |  |  |  |  |  |  |  |  |  |  |  |  |  |  |  |  |  |  |  |  |  |  | |  |  |  |  |  |  |  |
| Higher Income | 2 | 12 | 1 |  | + |  |  | Ø |  |  |  |  | Ø | Ø | - | Ø |  |  | Ø |  |  | Ø | Ø |  |  | + |  | Ø | Ø | Ø |  |  |  | Ø |  |  |  |  |  |  | |  |  | Ø |  |  |  |  |
| Household wealth (2^nd^ highest vs. highest) | 1 | 0 | 0 |  |  |  |  |  |  |  |  |  |  |  |  |  |  |  |  |  |  |  |  |  |  | + |  |  |  |  |  |  |  |  |  |  |  |  |  |  | |  |  |  |  |  |  |  |
| Public assistance household | 0 | 1 | 0 |  |  |  |  |  |  |  |  |  |  |  |  |  |  |  |  |  |  |  |  |  |  |  |  |  |  | Ø |  |  |  |  |  |  |  |  |  |  | |  |  |  |  |  |  |  |
| **Organization** |  |  |  |  |  |  |  |  |  |  |  |  |  |  |  |  |  |  |  |  |  |  |  |  |  |  |  |  |  |  |  |  |  |  |  |  |  |  |  |  | |  |  |  |  |  |  |  |
| Health insurance | 1 | 1 | 0 |  |  |  |  |  |  |  |  |  |  |  |  |  |  |  |  |  |  | Ø |  |  |  |  |  |  |  |  |  |  |  |  |  |  |  |  |  |  | |  |  | + |  |  |  |  |
| Regular medical doctor | 0 | 2 | 0 |  |  |  |  |  |  |  |  |  |  |  |  |  |  |  |  |  |  | Ø | Ø |  |  |  |  |  |  |  |  |  |  |  |  |  |  |  |  |  | |  |  |  |  |  |  |  |
| Availability | 1 | 1 | 0 |  |  |  |  |  |  |  |  |  |  | + | Ø |  |  |  |  |  |  |  |  |  |  |  |  |  |  |  |  |  |  |  |  |  |  |  |  |  | |  |  |  |  |  |  |  |
| Acceptability | 0 | 2 | 0 |  |  |  |  |  |  |  |  |  |  | Ø | Ø |  |  |  |  |  |  |  |  |  |  |  |  |  |  |  |  |  |  |  |  |  |  |  |  |  | |  |  |  |  |  |  |  |
| Accessibility | 0 | 2 | 0 |  |  |  |  |  |  |  |  |  |  | Ø | Ø |  |  |  |  |  |  |  |  |  |  |  |  |  |  |  |  |  |  |  |  |  |  |  |  |  | |  |  |  |  |  |  |  |
| Social support | 2 | 3 | 1 |  |  |  |  |  |  |  |  |  | + | + | Ø |  |  |  |  |  |  |  |  |  |  |  |  |  |  |  | Ø |  |  |  | Ø- |  |  |  |  |  | |  |  |  |  |  |  |  |
| ***Need*** |  |  |  |  |  |  |  |  |  |  |  |  |  |  |  |  |  |  |  |  |  |  |  |  |  |  |  |  |  |  |  |  |  |  |  |  |  |  |  |  | |  |  |  |  |  |  |  |
| **Mental health** |  |  |  |  |  |  |  |  |  |  |  |  |  |  |  |  |  |  |  |  |  |  |  |  |  |  |  |  |  |  |  |  |  |  |  |  |  |  |  |  | |  |  |  |  |  |  |  |
| Severity of depression | 7 | 7 | 0 |  | Ø |  |  |  |  |  |  |  |  |  |  |  |  |  |  |  |  | Ø | + |  |  |  |  |  |  | Ø |  | + | + | + |  | + | + |  |  | Ø | | Ø |  | + |  |  | Ø | Ø |
| Longer duration of episode | 6 | 5 | 0 |  |  |  |  |  |  |  |  |  |  |  |  |  |  |  |  |  |  | + | + |  |  |  |  | Ø | Ø | Ø |  |  |  | + | + | + |  |  |  | Ø | Ø |  |  |  |  |  |  | + |
| > 1 major depressive episodes | 3 | 5 | 0 |  |  |  |  |  |  |  |  |  | + |  |  |  |  |  |  |  |  |  |  |  |  |  |  | Ø | Ø | + |  |  |  | Ø | Ø |  |  |  |  | + | Ø |  |  |  |  |  |  |  |
| Endogenous subtype | 1 | 0 | 0 |  |  |  |  |  |  |  |  |  |  |  |  |  |  |  |  |  |  |  |  |  |  |  |  |  |  |  |  |  |  | + |  |  |  |  |  |  |  |  |  |  |  |  |  |  |
| Past year suicide attempt | 1 | 4 | 0 |  |  |  |  |  |  |  |  |  |  | + | Ø | Ø |  |  | Ø |  |  |  |  |  |  |  |  |  |  | Ø |  |  |  |  |  |  |  |  |  |  | |  |  |  |  |  |  |  |
| Suicide thoughts/ideation | 4 | 4 | 0 |  |  |  |  |  |  |  |  |  | + |  |  |  |  |  | Ø |  |  |  |  |  |  |  |  | Ø | Ø | Ø |  |  |  | + | + | + |  |  |  |  | |  |  |  |  |  |  |  |
| Lack of interest | 1 | 0 | 0 |  |  |  |  |  |  |  |  |  |  |  |  |  |  |  |  |  |  |  |  |  |  |  |  |  |  |  |  |  |  |  | + |  |  |  |  |  | |  |  |  |  |  |  |  |
| Anhedonia/depressed mood | 1 | 1 | 0 |  |  |  |  |  |  |  |  |  |  |  |  |  |  |  |  |  |  |  |  |  |  |  |  |  |  |  |  |  |  | Ø | + |  |  |  |  |  | |  |  |  |  |  |  |  |
| Lack of energy/ tiredness/ fatigue | 1 | 2 | 0 |  |  |  |  |  |  |  |  |  |  |  |  |  |  |  |  |  |  |  |  |  |  |  |  |  |  |  |  |  |  | Ø | Ø | + |  |  |  |  | |  |  |  |  |  |  |  |
| Guilt | 1 | 1 | 0 |  |  |  |  |  |  |  |  |  |  |  |  |  |  |  |  |  |  |  |  |  |  |  |  |  |  |  |  |  |  | Ø | + |  |  |  |  |  | |  |  |  |  |  |  |  |
| appetite or weight loss | 1 | 1 | 0 |  |  |  |  |  |  |  |  |  |  |  |  |  |  |  |  |  |  |  |  |  |  |  |  |  |  |  |  |  |  | + | Ø |  |  |  |  |  | |  |  |  |  |  |  |  |
| appetite or weight gain | 0 | 2 | 0 |  |  |  |  |  |  |  |  |  |  |  |  |  |  |  |  |  |  |  |  |  |  |  |  |  |  |  |  |  |  | Ø | Ø |  |  |  |  |  | |  |  |  |  |  |  |  |
| Insomnia | 2 | 0 | 0 |  |  |  |  |  |  |  |  |  |  |  |  |  |  |  |  |  |  |  |  |  |  |  |  |  |  |  |  |  |  | + | + |  |  |  |  |  | |  |  |  |  |  |  |  |
| Hypersomnia | 1 | 1 | 0 |  |  |  |  |  |  |  |  |  |  |  |  |  |  |  |  |  |  |  |  |  |  |  |  |  |  |  |  |  |  | Ø | + |  |  |  |  |  | |  |  |  |  |  |  |  |
| Trouble concentrating/ slowed thinking | 3 | 0 | 0 |  |  |  |  |  |  |  |  |  |  |  |  |  |  |  |  |  |  |  |  |  |  |  |  |  |  |  |  |  |  | + | + | + |  |  |  |  | |  |  |  |  |  |  |  |
| Delusions | 1 | 0 | 0 |  |  |  |  |  |  |  |  |  |  |  |  |  |  |  |  |  |  |  |  |  |  |  |  |  |  |  |  |  |  | + |  |  |  |  |  |  | |  |  |  |  |  |  |  |
| Hallucinations | 1 | 0 | 0 |  |  |  |  |  |  |  |  |  |  |  |  |  |  |  |  |  |  |  |  |  |  |  |  |  |  |  |  |  |  | + |  |  |  |  |  |  | |  |  |  |  |  |  |  |
| Agitation/psychomotor change | 0 | 2 | 0 |  |  |  |  |  |  |  |  |  |  |  |  |  |  |  |  |  |  |  |  |  |  |  |  |  |  |  |  |  |  | Ø | Ø |  |  |  |  |  | |  |  |  |  |  |  |  |
| Retardation | 0 | 1 | 0 |  |  |  |  |  |  |  |  |  |  |  |  |  |  |  |  |  |  |  |  |  |  |  |  |  |  |  |  |  |  | Ø |  |  |  |  |  |  | |  |  |  |  |  |  |  |
| Low perceived mental health | 2 | 1 | 0 |  |  |  |  |  |  |  |  |  |  | + | Ø |  |  |  |  |  |  |  |  |  |  |  |  |  |  | + |  |  |  |  |  |  |  |  |  |  | |  |  |  |  |  |  |  |
| (subjective) disability | 2 | 4 | 0 |  |  |  |  |  |  |  |  |  |  |  |  |  |  |  |  |  |  |  |  |  |  |  |  | Ø | Ø | Ø |  | Ø |  |  |  | + | + |  |  |  | |  |  |  |  |  |  |  |
| Impairment in (social) functioning caused by mental illness | 1 | 3 | 0 |  |  |  |  |  |  |  |  |  |  |  |  | Ø |  |  |  |  |  | Ø | Ø |  |  |  |  |  |  |  |  |  |  | + |  |  |  |  |  |  | |  |  |  |  |  |  |  |
| Extra effort days (subjective) | 1 | 0 | 0 |  |  |  |  |  |  |  |  |  |  |  |  |  |  |  |  |  |  |  |  |  |  |  |  |  |  | + |  |  |  |  |  |  |  |  |  |  | |  |  |  |  |  |  |  |
| Absent from work | 1 | 0 | 0 |  |  |  |  |  |  |  |  |  |  |  |  |  |  |  |  |  |  |  |  |  |  |  |  |  |  |  |  |  |  |  | + |  |  |  |  |  | |  |  |  |  |  |  |  |
| Poor performance evaluation | 1 | 0 | 0 |  |  |  |  |  |  |  |  |  |  |  |  |  |  |  |  |  |  |  |  |  |  |  |  |  |  |  |  |  |  |  | + |  |  |  |  |  | |  |  |  |  |  |  |  |
| Tardy to work/left work early/missed project deadlines/required to redo work | 0 | 1 | 0 |  |  |  |  |  |  |  |  |  |  |  |  |  |  |  |  |  |  |  |  |  |  |  |  |  |  |  |  |  |  |  | Ø |  |  |  |  |  | |  |  |  |  |  |  |  |
| **Comorbidity** |  |  |  |  |  |  |  |  |  |  |  |  |  |  |  |  |  |  |  |  |  |  |  |  |  |  |  |  |  |  |  |  |  |  |  |  |  |  |  |  | |  |  |  |  |  |  |  |
| Psychiatric comorbidity | 1 | 3 | 0 |  | + |  |  |  | Ø |  |  |  |  |  |  | Ø |  |  |  |  |  |  |  |  |  |  |  |  |  | Ø |  |  |  |  |  |  |  |  |  |  | |  |  |  |  |  |  |  |
| Substance dependence/use disorder | 1 | 6 | 0 |  |  |  |  |  |  |  |  |  |  | Ø | Ø | Ø |  |  |  |  |  |  |  |  | + |  |  |  |  | Ø |  |  |  | Ø |  |  |  |  |  |  | | Ø |  |  |  |  |  |  |
| Anxiety disorder | 7 | 4 | 0 |  |  |  |  |  |  |  |  |  |  | + | + | + |  |  |  |  |  |  |  |  |  |  | + | Ø | + | Ø |  |  |  | +Ø |  |  | + |  |  |  | |  |  |  |  |  |  | Ø |
| Chronic depression/dysthymia | 0 | 5 | 0 |  |  |  |  |  |  |  |  |  |  |  |  |  |  |  |  |  |  |  |  |  |  |  |  | Ø | Ø | Ø |  |  |  | Ø | Ø |  |  |  |  |  | |  |  |  |  |  |  |  |
| Chronic somatic disorder | 5 | 6 | 0 | + | Ø |  |  |  |  |  |  |  | + | + | + | + |  |  |  |  |  | Ø | Ø |  |  |  |  |  |  | Ø |  | Ø |  |  |  |  |  |  |  |  | |  |  |  |  |  |  | Ø |
| Painful physical symptoms | 0 | 1 | 1 | - |  | Ø |  |  |  |  |  |  |  |  |  |  |  |  |  |  |  |  |  |  |  |  |  |  |  |  |  |  |  |  |  |  |  |  |  |  | |  |  |  |  |  |  |  |
| Somatic symptoms | 0 | 2 | 0 |  |  |  |  |  |  |  |  |  |  | Ø | Ø |  |  |  |  |  |  |  |  |  |  |  |  |  |  |  |  |  |  |  |  |  |  |  |  |  | |  |  |  |  |  |  |  |
| General health | 0 | 4 | 0 |  |  |  |  |  |  |  |  |  | Ø |  |  |  |  |  |  |  |  | Ø | Ø |  |  |  |  |  |  |  |  | Ø |  |  |  |  |  |  |  |  | |  |  |  |  |  |  |  |
| ***Contextual Characteristics*** |  |  |  |  |  |  |  |  |  |  |  |  |  |  |  |  |  |  |  |  |  |  |  |  |  |  |  |  |  |  |  |  |  |  |  |  |  |  |  |  | |  |  |  |  |  |  |  |
| Urban (vs. rural) | 0 | 5 | 0 |  | Ø |  |  | Ø |  |  |  |  |  |  |  | Ø |  |  | Ø |  |  |  |  |  |  |  |  |  |  | Ø |  |  |  |  |  |  |  |  |  |  | |  |  |  |  |  |  |  |
| Canada (vs. Europe/US) | 0 | 2 | 0 |  |  |  | Ø |  |  |  |  |  |  |  |  |  |  |  |  |  |  | Ø |  |  |  |  |  |  |  |  |  |  |  |  |  |  |  |  |  |  | |  |  |  |  |  |  |  |
| Different regions in the USA | 1 | 1 | 0 |  |  |  |  |  |  |  |  |  |  |  |  |  |  |  | + |  |  |  | |  |  |  |  |  |  |  |  |  |  |  | Ø |  |  |  |  |  | |  |  |  |  |  |  |  |

*Notes.* If adjusted and unadjusted results were reported in the same study for the same variable, only the adjusted results were listed in the table. + = significant positive association between characteristic and help-seeking behaviour; - = significant negative association between characteristic and help-seeking behaviour; Ø = no significant association between characteristic and help-seeking behaviour; x = significant differences between different groups; W = white; B = black; C = Canada; U = US; a-z were used to represent different data sets; a = ESEMeD (European Study of the Epidemiology of Mental Disorders); e: CCHS-1.1 (Canadian Community Health Survey on Mental Health and Well Being); f = CCHS-1.2; g =NESARC (National Epidemiologic Survey on Alcohol and Related Conditions); j = NSDUH (National Survey on Drug Use and Health); k = NCS (National Comorbidity Survey); l = OHS (Ontario Health Study); m = EHS (Estonian Health Survey); v = NCS-R (National Comorbidity Survey – Replication); w = NLAAS (National Latino and Asian American Study); x = NSAL (National Survey of American Life).
